# A Comparison Study of Vitamin D Deficiency among Older Adults in China and the United States

**DOI:** 10.1038/s41598-019-56297-y

**Published:** 2019-12-23

**Authors:** Jia Wei, Anna Zhu, John S. Ji

**Affiliations:** 1 Environmental Research Center, Duke Kunshan University, Kunshan, Jiangsu China; 2 Nicholas School of the Environment, Duke University, Durham, North Carolina USA

**Keywords:** Nutrition, Epidemiology

## Abstract

Vitamin D deficiency is a common health concern worldwide. We aim to compare the prevalence of vitamin D deficiency among older adults (65+) in China and the United States (US). We used data from the 2011 wave of Chinese Longitudinal Healthy Longevity Survey (CLHLS) in China (n = 2180), and 2011–2014 National Health and Nutrition Examination Survey (NHANES) in the US (n = 2283). Serum 25-hydroxyvitamin D [25(OH)D] was measured and a level of under 30/50 nmol/L was defined as vitamin D severe deficiency/deficiency. Risk factors of vitamin D deficiency were examined by multivariate regression models. We found that the mean 25(OH)D concentration was lower in China than in the US (45.1 vs. 83.5 nmol/L), with Chinese elderly lower than American elderly for every age group. 70.3% in China and 17.4% in the US were considered as vitamin D deficiency (30.6% and 3.4% were considered as severe deficiency). Older age, females, ethnic minorities, higher household income, self-rated “very bad” health, and never drinkers, were statistically significant in predicting lower serum 25(OH)D levels in China. In the US, males, ethnic minorities, lower income, self-rated “very bad” health, physically inactive, overweight, and obese were related to lower serum 25(OH)D levels. Our findings suggest that different interventional strategies are needed to improve vitamin D deficiency and its associated negative health outcomes in China and the US.

## Introduction

Vitamin D deficiency is a serious health condition worldwide. Vitamin D is essential for human bone health, and severe vitamin D deficiency increases the risk of many diseasesincluding osteomalacia, osteoporosis, muscle weakness, hip fractures, diabetes, cancer, heart disease, arthritis, and poor general health in the elderly^1–3^. The aging process is considered a risk of vitamin D deficiency, because of debilitated ability to synthesize vitamin D from sunlight, activation of vitamin D in the kidney, and less outdoor exercise and activity^4^.

The US National Academy of Medicine (formerly the Institute of Medicine) considers a serum 25-hydroxyvitamin D (25(OH)D) level of at least 50 nmol/L as the adequate exposure to vitamin D to maintain bone health. Individuals with levels less than 30 nmol/L are considered as severe deficient^5^. In this study, we aim to report serum 25(OH)D concentrations in China and the United States (US) using data from the CLHLS (Chinese Longitudinal Healthy Longevity Survey) and the NHANES (US National Health and Nutrition Examination Survey), which are nationally representative surveys of older adults from both countries.

## Results

CLHLS had a larger proportion of older adults aged 80 years and older (67.0%), with a mean age of 85.9 years old, compared to 26.7% in NHANES with a mean age of 73.3 years old (Table 1). In China, only 37.0% of the participants had some formal education, while in the US, around 70% had a high school education and above. More Chinese participants were widowed (57.4%) than US participants (26.8%). More Chinese participants rated their health condition as “good” (35.5%) than US participants (23.1%), and fewer rated as “bad” in China (11.2%) than in the US (23.7%). Smoking and drinking behaviors were more common in the US than in China. More Chinese participants never smoked (72.6%) or drank any alcohol (76.6%) than US participants (50.0% and 18.0%, respectively). The Chinese sample was more physically inactive than the US sample (80.3% versus 41.2% do not have physical activity). China had much more underweight participants (24.0%) than the US (1.7%), while the US had much more overweight and obese participants (35.4% and 34.1%, respectively) than China (10.6% and 3.1%, respectively).

**Table 1:** Baseline characteristics of the CLHLS and NHANES participants.

China (CLHLS 2011)		US (NHANES 2011–2014)	
	n,%		n,%
Total	2,180 (100)	Total	2,283 (100)
Age (mean ± SD)	85.9 ± 12.0	Age (mean ± SE)	73.3±0.1
**Age group**		**Age group**	
65–69	247 (11.3)	65–69	703 (30.8)
70–74	249 (11.4)	70–74	592 (25.9)
75–79	223 (10.2)	75–79	379 (16.6)
80+	1,461 (67.0)	80+	609 (26.7)
**Gender**		**Gender**	
Male	991 (45.5)	Male	1,111 (48.6)
Female	1,189 (54.5)	Female	1,172 (51.4)
**Race**		**Race/Ethnicity**	
Han Chinese	1,970 (90.4)	Mexican American	174 (7.7)
Ethnic minorities	158 (7.3)	Other Hispanics	197 (8.6)
Missing	52 (2.4)	Non-Hispanic White	1,210 (53.0)
		Non-Hispanic Black	464 (20.3)
		Non-Hispanic Asian	202(8.9)
		Other races	36 (1.6)
**Education**		**Education**	
No formal education	1,353 (62.1)	Less than 9th grade	353 (15.5)
Formal education	806 (37.0)	9–11th grade (Includes 12th grade with no diploma)	329 (14.4)
Missing	21 (1.0)	High school graduate/GED or equivalent	528 (23.1)
		Some college or AA degree	580 (25.4)
		College graduate or above	488 (21.4)
		Missing	5 (0.2)
**Marital Status**		**Marital Status**	
Married	804 (36.9)	Married	1,250 (54.8)
Separated	39 (1.8)	Separated	48 (2.1)
Divorced	5 (0.2)	Divorced	270 (11.8)
Widowed	1,252 (57.4)	Widowed	613 (26.8)
Never married	23 (1.1)	Never married	100 (4.4)
Missing	57 (2.6)	Missing	2 (0.1)
**Household income**		**Income(PIR)**	
Tertile1 (0–6,000 RMB)	719 (33.0)	0–1.85	974 (42.7)
Tertile2 (6,200–20,000 RMB)	698 (32.0)	1.86–3.50	550 (24.1)
Tertile3 (21,000-more than 100,000 RMB)	623 (28.6)	>3.51	558 (24.4)
Missing	140 (6.4)	Missing	201 (8.8)
**Health condition**		**Health condition**	
Very good	106 (4.9)	Very good	160 (7.0)
Good	774 (35.5)	Good	527 (23.1)
Fair	839 (38.5)	Fair	840 (36.8)
Bad	243 (11.2)	Bad	540 (23.7)
Very Bad	18 (0.8)	Very Bad	104 (4.5)
Missing	200 (9.2)	Missing	112 (4.9)
**Smoking status**		**Smoking status**	
Never smoker	1,582 (72.6)	Never smoker	1,142 (50.0)
Former smoker	176 (8.1)	Past smoker	908 (39.8)
Current smoker	356 (16.3)	Current smoker	231 (10.1)
Missing	66 (3.0)	Missing	2 (0.1)
**Drinking status**		**Drinking status**	
Never drinker	1,670 (76.6)	Never drinker	411 (18.0)
Former drinker	128 (5.9)	Past drinker	631 (27.6)
Current drinker	322 (14.8)	Current drinker	1,104 (48,4)
Missing	60 (2.8)	Missing	137 (6.0)
**Physical activity**		**Physical activity**	
Yes	323 (14.8)	Yes	1,343 (58.8)
No	1,750 (80.3)	No	940 (41.2)
Missing	107 (4.9)		
**Sleep duration**		**Sleep duration**	
<6 h	383 (17.6)	<6 h	286 (12.5)
6–9 h	1,308 (60.0)	6–9 h	1,872 (82.0)
>9 h	475 (21.8)	>9 h	120 (5.3)
Missing	14 (0.6)	Missing	5 (0.2)
**BMI**		**BMI**	
Underweight (0–18.5)	524 (24.0)	Underweight (0–18.5)	38 (1.7)
Normal (18.5–25)	1,243 (57.0)	Normal (18.5–25)	606 (26.5)
Overweight (25–30)	231 (10.6)	Overweight (25–30)	808 (35.4)
Obese (> = 30)	67 (3.1)	Obese (> = 30)	778 (34.1)
Missing	115 (5.3)	Missing	53 (2.3)
		**VD supplement**	
		No	1110 (48.6)
		Yes	1173 (51.4)

There was a large difference in the serum level of 25(OH)D between China and the US. The mean serum 25(OH)D level in China was much lower (45.1 nmol/L) than in the US (83.5 nmol/L) (Table 2). In China, serum 25(OH)D level decreased by age and was significantly higher in males (p < 0.0001), while in the US, we saw an increasing trend with age and was significantly higher in females (p < 0.0001). In both countries, serum 25(OH)D level differed by races. It was significantly higher in Han Chinese than in ethnic minorities in China (P = 0.0357), and higher in non-Hispanic whites than other races (p < 0.0001). In the US, higher serum 25(OH)D levels were associated with higher educational levels and family income. In China, older adults with formal education, and lowest tertile of household income had higher serum 25(OH)D level. In the US, serum 25(OH)D significantly decreased with the worse health condition (p < 0.0001). In China, those who rated their health condition as “Very bad” had the lowest serum 25(OH)D concentration.

**Table 2:** Serum 25(OH)D levels by baseline characteristics among CLHLS and NHANES participants.

China (CLHLS 2011)			US (NHANES 2011–2014)		
	Mean (SE)	P value		Mean (SE)	P value
Total	45.1 (0.7)		Total	83.5 (1.0)	
**Month of blood draw**		<0.0001	**Season of blood draw**		<0.0001
May	40.9 (1.5)		Summer	85.6 (1.1)	
June	43.1 (0.9)		Winter	80.6 (1.7)	
July	48.2 (1.2)				
August	66.6 (5.7)				
September	61.3 (3.6)				
**Age group**		<0.0001	**Age group**		0.03
65–69	46.8 (1.5)		65–69	80.1 (1.7)	
70–74	45.9 (1.3)		70–74	84.9 (1.6)	
75–79	45.2 (1.1)		75–79	84.4 (2.0)	
80+	41.6 (0.7)		80+	85.9 (1.6)	
**Gender**		<0.0001	**Gender**		<0.0001
Male	50.4 (1.0)		Male	78.2 (0.9)	
Female	40.1 (0.8)		Female	87.8 (1.6)	
**Race**		0.0357	**Race/Ethnicity**		<0.0001
Han Chinese	45.7 (0.7)		Mexican American	66.8 (2.9)	
Ethnic minorities	40.7 (1.7)		Other Hispanics	70.9 (3.0)	
Missing	39.0 (3.8)		Non-Hispanic White	86.1 (1.0)	
			Non-Hispanic Black	69.7 (1.6)	
			Non-Hispanic Asian	82.8 (2.7)	
			Other races	86.8 (2.3)	
**Education**		<0.0001	**Education**		0.005
No formal education	42.2 (0.9)		Less than 9th grade	74.1 (2.1)	
Formal education	47.4 (1.0)		9–11th grade (Includes 12th grade with no diploma)	82.8 (2.2)	
Missing	44.6 (2.7)		High school graduate/GED or equivalent	84.1 (2.3)	
			Some college or AA degree	82.6 (1.7)	
			College graduate or above	87.3 (1.5)	
			Missing	87.4	
**Marital Status**		<0.0001	**Marital Status**		0.1
Married	46.7 (0.9)		Married	84.0 (1.1)	
Separated	45.2 (3.5)		Separated	71.7 (3.6)	
Divorced	51.1 (4.6)		Divorced	83.1 (2.3)	
Widowed	41.7 (0.9)		Widowed	83.3 (2.1)	
Never married	54.3 (9.1)		Never married	80.9 (2.2)	
Missing	41.6 (3.5)		Missing	50	
**Household income**		0.0073	**Income(PIR)**		<0.0001
Tertile1 (0–6,000 RMB)	47.4 (1.3)		0–1.85	78.7 (1.6)	
Tertile2 (6,200–20,000 RMB)	44.4 (1.0)		1.86–3.50	83.8 (1.9)	
Tertile3 (21,000- > 100,000 RMB)	44.5 (1.2)		>3.51	88.1 (1.5)	
Missing	39.4 (2.1)		Missing	81.6 (3.8)	
** Health condition**		<0.0001	**Health condition**		<0.0001
Very good	45.5 (2.3)		Very good	86.6 (3.2)	
Good	45.9 (1.1)		Good	89.3 (2.4)	
Fair	45.4 (0.9)		Fair	82.2 (1.4)	
Bad	43.6 (1.9)		Bad	77.5 (2.0)	
Very Bad	32.7 (3.8)		Very Bad	74.7 (4.5)	
Missing	36.9 (3.0)		Missing	82.0 (3.6)	
**Smoking status**		<0.0001	**Smoking status**		0.03
Never smoker	43.0 (0.7)		Never smoker	84.4 (1.3)	
Former smoker	49.4 (2.1)		Past smoker	84.4 (1.0)	
Current smoker	51.7 (1.6)		Current smoker	74.6 (3.9)	
Missing	40.8 (2.6)		Missing	41.8	
**Drinking status**		<0.0001	**Drinking status**		0.8
Never drinker	43.2 (0.7)		Never drinker	83.5 (2.4)	
Former drinker	42.8 (2.4)		Past drinker	84.1 (1.9)	
Current drinker	55.8 (1.9)		Current drinker	83.3 (1.2)	
Missing	42.8 (3.0)		Missing	82.9 (3.8)	
**Physical activity**		<0.0001	**Physical activity**		0.001
Yes	46.0 (1.4)		Yes	86.0 (1.2)	
No	45.0 (0.8)		No	79.4 (1.5)	
Missing	44.2 (3.3)				
**Sleep duration**		<0.0001	**Sleep duration**		0.5
<6 h	46.0 (1.3)		<6 h	79.6 (2.7)	
6–9 h	45.2 (0.8)		6–9 h	84.1 (1.0)	
>9 h	43.7 (1.7)		>9 h	80.1 (4.7)	
Missing	41.4 (7.9)		Missing	59.5	
**BMI**		<0.0001	**BMI**		<0.0001
Underweight (0–18.5)	46.4 (1.8)		Underweight (0–18.5)	91.9 (5.7)	
Normal (18.5–25)	44.8 (0.8)		Normal (18.5–25)	90.4 (1.8)	
Overweight (25–30)	46.0 (1.8)		Overweight (25–30)	83.3 (1.5)	
Obese (> = 30)	44.7 (3.1)		Obese (> = 30)	77.9 (1.5)	
Missing	37.6 (2.8)		Missing	84.1 (2.7)	
			**VD supplement**		<0.0001
			No	67.9 (1.2)	
			Yes	94.7 (1.0)	

In China, current smokers and current drinkers had significantly higher serum 25(OH)D levels. In the US, serum 25(OH)D level was lower in current smokers, but was not affected by drinking behavior. In both countries, older adults having physical activities had significantly higher serum 25(OH)D level (p = 0.0001 and 0.001, respectively). As for BMI, older adults with higher BMI had lower serum 25(OH)D level in the US (p < 0.0001), but the similar trend did not appear in China.

There were different predictors of serum 25(OH)D between China and the US (Table 3). In China, participants who were aged 80 and over, females, ethnic minorities, with higher household income, bad at self-rated health, and current drinkers, had lower serum 25(OH)D level. In the US, participants who were younger than 70, males, Mexican American, Mexican American, other Hispanics, had lower income, bad at self-rated health, did not have physical activity, were overweight, and obese, had lower serum 25(OH)D level. Similar findings were observed for the risk factors of vitamin D deficiency. Additionally, seasonal variation of serum 25(OH)D was significant in CLHLS.

**Table 3:** Linear regression predicting serum 25(OH)D level and logistic regression predicting vitamin D deficiency in China and the US.

China (CLHLS 2011)*					US (NHANES 2011–2014)**				
Predictors	Coefficient (95%CI)	p value	OR (95%CI)	p value	Predictors	Coefficient (95%CI)	p value	OR (95%CI)	p value
**Month of blood draw**					Season of blood draw				
**May**	**Ref**		**Ref**		**Winter**	**Ref**		**Ref**	
June	5.19 (2.06, 8.32)	0.001	1.59 (1.00, 2.53)	0.050	Summer	3.58 (−1.26, 8.43)	0.10	0.68 (0.49, 0.99)	0.049
July	12.93 (9.14, 16.71)	<0.001	3.16 (1.90, 5.25)	<0.001					
August	29.69 (18.45, 40.93)	<0.001	19.14 (6.68, 54.86)	<0.001					
September	23.86 (15.48, 32.23)	<0.001	8.35 (2.57, 27.10)	<0.001					
**Age group**					**Age group**				
**65–69**	**Ref**		**Ref**		**65–69**	**Ref**		**Ref**	
70–74	−0.73 (−4.31, 2.85)	0.689	1.12 (0.68, 1.83)	0.653	70–74	4.59 (0.57, 8.62)	0.03	0.61 (0.38, 0.99)	0.046
75–79	−1.90 (−5.50, 1.70)	0.300	0.80 (0.47, 1.35)	0.402	75–79	5.19 (0.13, 10.25)	0.04	0.62 (0.37, 1.05)	0.07
80+	−4.53 (−8.05, −1.02)	0.012	0.66 (0.40, 1.10)	0.113	80+	3.33 (−1.36, 8.03)	0.2	0.77 (0.50, 1.21)	0.3
**Gender**					**Gender**				
**Male**	**Ref**		**Ref**		**Male**	**Ref**		**Ref**	
Female	−7.16 (−10.51, −3.82)	<0.001	0.40 (0.25, 0.64)	<0.001	Female	12.13 (8.31, 15.94)	<0.0001	1.05 (0.68, 1.64)	0.8
**Race**					**Race/Ethnicity**				
**Han Chinese**	**Ref**		**Ref**		Mexican American	−9.57 (−16.36, −2.79)	0.01	2.92 (1.59, 5.38)	0.0005
Ethnic minorities	−4.77 (−8.72, −0.82)	0.018	0.48 (0.24, 0.96)	0.037	Other Hispanics	−11.39 (−20.13, −2.65)	0.01	2.19 (1.02, 4.69)	0.04
					**Non-Hispanic White**	**Ref**		**Ref**	
					Non-Hispanic Black	−13.00 (−16.78, −9.23)	<0.0001	3.43 (2.35, 5.03)	<0.0001
					Non-Hispanic Asian	−6.77 (−12.83, −0.71)	0.03	2.25(1.16, 4.35)	0.02
					Other races	8.13 (−2.17, 18.43)	0.1	0.84(0.16, 4.52)	0.8
**Education**					**Education**				
**No formal education**	**Ref**		**Ref**		**Less than 9th grade**	**Ref**		**Ref**	
Formal education	0.05 (−2.86, 2.97)	0.971	1.05 (0.70, 1.58)	0.813	9–11th grade (Includes 12th grade with no diploma)	2.48 (−2.29, −7.26)	0.30	1.20 (0.67, 2.13)	0.5
					High school graduate/GED or equivalent	1.16 (−5.23, 7.55)	0.70	1.58 (0.83, 3.03)	0.2
					Some college or AA degree	−2.00 (−8.06, 4.06)	0.50	1.02 (0.55, 1.90)	0.9
					College graduate or above	0.26 (−6.28, 6.81)	0.90	1.06 (0.48, 2.37)	0.9
**Marital Status**					**Marital Status**				
**Married**	**Ref**		**Ref**		**Married**	**Ref**		**Ref**	
Separated	−2.14 (−9.34, 5.07)	0.561	0.57 (0.15, 2.17)	0.414	Separated	−6.57 (−14.40, 1.26)	0.10	1.32 (0.62, 2.80)	0.5
Divorced	3.32 (−4.07, 10.71)	0.379	1.72 (0.22, 13.54)	0.606	Divorced	−0.29 (−6.10, 5.52)	0.90	1.32 (0.67, 2.62)	0.4
Widowed	−0.49 (−3.17, 2.18)	0.717	0.88 (0.59, 1.31)	0.529	Widowed	−1.98 (−6.55, 2.59)	0.40	1.44 (0.86, 2.41)	0.2
Never married	−3.09 (−18.49, 12.32)	0.694	0.34 (0.11, 1.09)	0.068	Never married	−1.60 (−7.20, 3.98)	0.60	0.98 (0.59, 1.64)	0.9
**Household income**					**Income(PIR)**				
**Tertile1 (0–6,000RMB)**	**Ref**		**Ref**		**0–1.85**	**Ref**		**Ref**	
Tertile2 (6,200–20,000 RMB)	−4.56 (−7.44, −1.69)	0.002	0.63 (0.41, 0.96)	0.032	1.86–3.50	2.96 (−0.55, 6.48)	0.10	0.78 (0.54, 3.13)	0.2
Tertile3 (21,000- > 100,000RMB)	−4.48 (−7.83, −1.13)	0.009	0.52 (0.32, 0.83)	0.007	>3.51	7.02 (1.25, 12.79)	0.02	0.55 (0.30, 0.99)	0.048
**Health condition**					**Health condition**				
Very good	−0.43 (−5.48, 4.63)	0.869	0.65 (0.27, 1.57)	0.341	Very good	1.45 (−6.00, 8.91)	0.70	0.49 (0.86, 2.41)	0.1
Good	1.39 (−1.32, 4.10)	0.315	1.11 (0.76, 1.64)	0.590	Good	5.39 (0.34, 10.43)	0.04	1.32 (0.67, 2.62)	0.3
**Fair**	**Ref**		**Ref**		**Fair**	**Ref**		**Ref**	
Bad	−2.08 (−6.12, 1.96)	0.313	0.85 (0.48, 1.48)	0.558	Bad	−0.83 (−4.63, 2.96)	0.70	1.44 (0.86, 2.41)	0.4
Very Bad	−9.70 (−14.10, −5.31)	<0.001	0.29 (0.076, 1.08)	0.065	Very Bad	−2.47 (−12.94, 8.00)	0.04	0.98 (0.59, 1.64)	0.5
**Smoking status**					**Smoking status**				
**Never smoker**	**Ref**		**Ref**		**Never smoker**	**Ref**		**Ref**	
Former smoker	2.78 (−2.87, 8.42)	0.334	1.23 (0.60, 2.52)	0.577	Former smoker	3.23 (−0.34, 6.79)	0.07	1.03 (0.74, 1.44)	0.9
Current smoker	0.68 (−3.30, 4.65)	0.739	1.31 (0.83, 2.08)	0.246	Current smoker	−3.91 (−10.52, 2.71)	0.20	1.95 (1.03, 3.70)	0.04
**Drinking status**					**Drinking status**				
**Never drinker**	**Ref**		**Ref**		**Never drinker**	**Ref**		**Ref**	
Former drinker	−2.82 (−8.63, 2.99)	0.341	0.89 (0.44, 1.80)	0.742	Former drinker	1.43 (−4.60, 7.47)	0.60	0.98 (0.63, 1.53)	0.9
Current drinker	8.70 (4.34, 13.05)	<0.001	1.74 (1.09, 2.79)	0.021	Current drinker	−3.29 (−7.89, 1.31)	0.20	1.40 (0.88, 2.23)	0.2
**Physical activity**					**Physical activity**				
Yes	0.27 (−3.04, 3.58)	0.872	1.32 (0.82, 2.11)	0.248	Yes	4.87 (1.61, 8.13)	0.01	0.63 (0.43, 0.94)	0.02
**No**	**Ref**		**Ref**		**No**	**Ref**		**Ref**	
**Sleep duration**					**Sleep duration**				
<6 h	−0.81 (−4.02, 2.40)	0.621	1.08 (0.64, 1.84)	0.766	<6 h	−0.28 (−6.96, 6.40)	0.90	0.92 (0.45, 1.85)	0.8
**6–9 h**	**Ref**		**Ref**		**6–9 h**	**Ref**		**Ref**	
>9 h	−2.73 (−6.39, 0.93)	0.144	0.69 (0.43, 1.12)	0.131	>9 h	−4.00 (−13.74, 5.74)	0.4	2.51 (1.46, 4.28)	0.0008
**BMI**					**BMI**				
Underweight (0–18.5)	1.31 (−2.16, 4.78)	0.460	1.16 (0.73, 1.84)	0.543	Underweight (0–18.5)	−4.74 (−23.03, 13.54)	0.6	0.84 (0.08, 8.47)	0.9
**Normal** **(18.5–25)**	**Ref**		**Ref**		**Normal (18.5–25)**	**Ref**		**Ref**	
Overweight (25–30)	3.00 (−0.56, 6.56)	0.099	1.71 (1.06, 2.75)	0.027	Overweight (25–30)	−6.45 (−10.10, −2.81)	0.001	1.48 (0.92, 2.38)	0.1
Obese (> = 30)	3.10 (−1.87, 8.06)	0.221	2.13 (0.94, 4.80)	0.069	Obese (> = 30)	−11.96 (−17.16, −6.77)	<0.0001	1.92 (1.20, 3.08)	0.007
					**vD supplement**				
					**No**	**Ref**		**Ref**	
					Yes	23.46 (20.71–26.22)	<0.0001	0.05 (0.03–0.09)	<0.0001

*All regression models were adjusted for month of blood draw, age, gender, ethnicity, education, marital status, household income, health condition, smoking and drinking status, physical activity, sleep duration, and BMI in the CLHLS analysis.

**All regression models were adjusted for season of sampling, age, gender, season, race/ethnicity, education, marital status, income, health condition, smoking and drinking status, physical activity, sleep duration, BMI, and vD supplement in the NHANES analysis.

## Discussion

Vitamin D is an essential micronutrient to human health, but there is no consensus on the optimal level of vitamin D. The US National Academy of Medicine recommended a serum 25(OH)D level of 50 nmol/L^5^. However, the US Endocrine Society recommended a higher level of 75 nmol/L for optimal health benefits^6^. Additionally, the European Society for Clinical and Economic Aspects of Osteoporosis and Osteoarthritis (ESCEO) recommended a minimal 25(OH)D level of 75 nmol/L for fragile elderly subjects^7^. The European Menopause and Andropause Society (EMAS) recommended elderly people to achieve serum 25(OH)D levels of 75–225 nmol/L^8^. In China, the Osteoporosis Committee of China Gerontological Society adopted the same standard as the National Academy of Medicine, with vitamin D deficiency defined as less than 30 nmol/L, insufficiency as 30–49.9 nmol/L, and sufficiency as more than 50 nmol/L^9^. However, all guidelines recommend or conclude that serum 25OHD concentrations below 25 nmol/l should be avoided in all subjects (of whatever age)^10^. In our study, 78 (3.4%) NHANES participants and 668 (30.6%) CLHLS participants were considered as severe vitamin D deficiency (<30 nmol/L). 398 NHANES participants (17.4%) and 1,572 CLHLS participants (70.7%) had a serum 25(OH)D level less than 50 nmol/L, indicating vitamin D deficiency or insufficiency.

Our study found lower serum 25(OH)D concentration among Chinese participants than US participants (45.1 vs 83.5 nmol/L), unexplained by possible confounding factors. The mean 25(OH)D concentration in our study was similar to some previous findings in China^11–16^. The mean serum 25(OH)D concentrations reported in prior studies in China are lower than our finding in the NHANES population, and also some prior studies in the US. Studies have been conducted to examine the 25(OH)D concentrations worldwide. In Europe, using the NIH-led international Vitamin D Standardized Program (VDSP) protocol, the mean 25(OH)D value of 5519 participants (mean age = 76.6) from Iceland was 57.0 nmol/L, and of 915 participants (mean age = 71.4) from the Netherlands was 64.7 nmol/L^17^. These results are higher than the value in China, but lower than the value in the US. Among these two cohorts, 8.4% and 4.6% were considered as severe deficiency (<30 nmol/L), which is similar to our US population (3.4%). In the Middle East countries, a study in Lebanon with 157 males and 286 females (mean age = 73) found a mean 25(OH)D value of 25.7 nmol/L. An Egypt study with elderly women (mean age = 76) found a mean 25(OH)D value of 37 nmol/L^18^. These results are more comparable to the results in China^19^.

Furthermore, we found there was a difference in predictors related to serum 25(OH)D levels between China and the US. In both the US and China, older adults had lower serum 25(OH)D level, consistent with prior findings^20,21^. The decline of serum 25(OH)D level in the aging process is linked to reduction in the skin production of vitamin D, calcium absorption of circulated 1,25(OH)2D, and renal production of 1,25(OH)2D^4^. At the same time, vitamin D supplement intake helps increase the serum 25(OH)D level, especially for the older adults. We believe the different serum 25(OH)D level over age between China and the US may be explained by the much higher vitamin D usage in the US than in China^20,22^.

We found a small gender difference in serum 25(OH)D level, observed both in China and the US. In China, females had lower serum 25(OH)D level, while males had lower concentrations in the US. In the US, a study of 2007–2010 NHANES reported no significant gender difference among adults aged 65 years and older^23^, while another study of 1998–2004 NHANES showed that males had significantly higher serum 25(OH)D level than females^24^. It may be possible that sun avoidance behavior was more prevalent in females, such as the use of sunscreen, protective clothes, and sunglasses^25,26^. Potential gender difference may also be caused by differences in hormone levels, lifestyle, and supplement usage^27^.

Income also affects serum 25(OH)D level. In the US, higher income was associated with higher serum 25(OH)D levels. The higher income group had more dietary supplement like vitamin D, and also more diverse nutrient sources of vitamin D^22^. However, in China, the older adults with higher household income were more likely to have lower serum 25(OH)D level, and this finding is different from the majority findings in other countries^28^. Because of the rapid urbanizing process, those of higher socioeconomic status may be more likely to live in cities and areas with higher population density, and hence reduce outdoor sunlight exposure. In a study using the 2010–2013 China National Nutrition and Health Survey (CNNHS), older adults aged 60 years and older living in large cities had a higher risk of vitamin D inadequacy than those living in general rural areas^20^. Furthermore, high air pollution in cities could also act as a barrier to UV light, although this pathway has not been clearly elucidated^29^.

Both CLHLS and NHANES presented that the older adults who were very bad at self-rated health had lower serum 25(OH)D level. Lower 25(OH)D level is related to bad health conditions. Prior studies have found a strong association between 25(OH)D and several health conditions, including delirium, high blood pressure, and lower total testosterone^30–32^, but there were also studies not supporting effects of 25(OH)D on diabetes, breast, prostate, and colorectal cancer^33–35^. On the other hand, bad health conditions may also lead to a reduced 25(OH)D level. For example, depressed individuals are often reluctant to engage in outdoor activities, and have reduced appetite, which can decrease 25(OH)D levels^36^. Hence, there might be a vicious cycle between lower vitamin D and bad health.

Being physically inactive and overweight were risk factors of lower serum 25(OH)D level in the US, but not in China. Several studies reported the positive association between physical activity and serum 25(OH)D level^37,38^. Physical activity increases sun exposure, and prevents loss of muscle strength and mass, which are the essential determinants of serum 25(OH)D level^38^. However, evidence found that vitamin D insufficiency was still common among people who were highly physically active in Germany^37^. This probably partly contributes to the difference between China and the US. Additionally, the relationship between a higher BMI and a lower serum 25(OH)D level has been well studied^39^. Overweight or obese people may have lower dietary supplement intake, reduced cutaneous synthesis, decreased intestinal absorption, and need more vitamin D intake according to the volumetric dilution model. In China, the percentage of overweight or obese older adults was much lower, and we did not see an association between BMI and serum 25(OH)D level, possibly due to a smaller sample size.

Our study used national representative samples and a diverse group of variables to assess. However, there were some limitations to our study as well. Firstly, different time of blood draw among the CLHLS and NHANES participants may bias our comparison analysis. In the CLHLS, the blood samples were collected from May to September, while in the NHANES, the samples were collected either in summer (May to October) or in winter (November to April). Serum 25(OH)D level is highly influenced by season due to sunlight availability, with higher concentrations in summer than in winter. Secondly, the measurement techniques of serum 25(OH)D used in the CLHLS differed from that in the NHANES. CLHLS applied enzyme-linked immunosorbent assay, while NHANES applied ultra-high performance liquid chromatography-tandem mass spectrometry (UHPLC-MS/MS)^40^. It was possible that different measurement techniques may contribute to a part of the difference in serum 25(OH)D between China and the US. However, it was unlikely to explain such a big difference in our study (54.1 nmol/L in China vs. 83.5 nmol/L in the US). Thirdly, we had information vitamin D supplement use in the NHANES but not in the CLHLS. Since vitamin D supplement use helps increase the serum 25(OH)D level, we were not sure that how much difference in serum 25(OH)D concentrations between NHANES and CLHLS was contributed by vitamin D supplement use. Fourthly, some confounding factors such as comorbidities, time spent outdoors, and residential areas were recorded differently or unavailable, thus were not adjusted for to make the two datasets more comparable. However, we did adjust for the general health condition variable as a proxy for comorbidity which was consistent in both datasets. Lastly, our study used a cross-sectional design, which could neither infer any causal relationships nor show the difference in the trends of serum 25(OH)D over the years. There is a possibility that changes in 25(OH)D levels may in turn affect people’s health conditions and behaviors. Further longitudinal studies could better inform the factors causally associated with 25(OH)D.

Our findings demonstrated a large difference in 25(OH)D levels between US and Chinese older adults, which has implications for further research on whether the current clinical guideline is appropriate for people of different age, race, and country of residence. In addition to study design differences between CLHLS and NHANES, the factor of race and ethnicity cannot be ignored. Many studies have shown racial differences contrasting vitamin D status. In Australia, UK, and Canada, immigrants from Asia, Middle East, and Africa had significantly lower 25(OH)D levels compared to the white population^41–43^. In our study, in NHANES population, non-Hispanic Asians and non-Hispanic blacks also showed lower serum 25(OH)D level than non-Hispanic whites. African Americans generally have lower levels of vitamin D than their white counterparts due to skin pigmentation reducing vitamin D production^44^. The difference in skin color could contribute to their different 25(OH)D concentration. The difference in culture and tradition could also explain the differences in vitamin D status. Studies have found that the consumption of vitamin D-enriched food showed ethnic differences, which could lead to different 25(OH)D levels^45,46^. Furthermore, differences in population genetics can play a part in vitamin D synthesis and metabolism^47^. Studies have found that group-specific component gene (GC) polymorphisms were associated with 25(OH)D levels, and allele frequencies were different among geographic regions worldwide^48^. For example, the GC1S haplotype which is related to a higher level of 25(OH)D is found to have the maximum frequency in white population, while the GC1F haplotype which is associated with lower vitamin D-binding protein levels, is more likely to be carried by Asians^49^. Vitamin D-associated genes may present different allele frequency between cohorts. Genetic determinants of vitamin D production and metabolism may be the underlying reasons why there is a racial difference in health responses in clinical guidelines, and this warrants further investigation. Therefore, future studies and clinical guidelines should take race/ethnicity into consideration when examining 25(OH)D levels in different populations.

## Method

### Study population

We used data from CLHLS and NHANES to compare serum 25(OH)D concentrations among the older population, aged 65 years or older. Both CLHLS and NHANES collected data through in-person interviews and blood samples.

The CLHLS was designed to explore the determinants of healthy longevity among Chinese older adults. Established in 1998, the CLHLS recruited new participants and conducted follow-up surveys in 2000, 2002, 2005, 2008, 2011, 2014, and 2018. The CLHLS has collected extensive data on the determinants of health, including demographic characteristics, socioeconomic status, lifestyle, physical capacity, cognitive function, and psychological well-being. The CLHLS used a multistage, stratified cluster sampling, and recruited participants from 22 out of 31 provinces in China. 631 cities and counties were randomly selected as the sample sites, which represent about 85% of the Chinese population. More details about sampling design and weight could be found elsewhere^50^. Our study used the 2011 wave of CLHLS. CLHLS collected blood samples in eight longevity regions with a higher proportion of older people^51^. A total of 2,439 participants were surveyed in this wave. We excluded participants if they had missing values of 25(OH)D concentration (n = 130), were younger than 65 years (n = 84), and were missing weight variable (n = 45). We had 2,180 participants in CLHLS for final analysis.

NHANES is a nationally representative survey of the US non-institutionalized population, identified through a complex sampling design with oversampling of lower socioeconomic status and ethnicities minorities. Household interviews were conducted by trained personnel to collect information on health and socio-demographic characteristics. Standardized physical examinations were conducted and blood samples were also drawn in mobile examination centers. In the current analysis, data from the 2011–2012 and 2013–2014 waves were merged. Older adults who were 65 years or older were included as the study sample, which yielded a total of 2556 participants. Then, those who had missing values for serum 25(OH)D measurements were excluded from the analysis (n = 273). The final sample consisted of 2283 older adults.

### Vitamin D measurement

Serum 25-hydroxyvitamin D (25(OH)D) was considered as the best biomarker of vitamin D status since it indicates sources of both sun exposure and diet^52^. In CLHLS, 25(OH)D was assessed by an enzyme-linked immunosorbent assay (Immunodiagnostic Systems Limited, Bolton, UK). The inter- and intraassay coefficients of variation were less than 10% and less than 8%, respectively^53^. In NHANES, ultra-high-performance liquid chromatography-tandem mass spectrometry (UHPLC-MS/MS) was utilized for the quantitative detection of 25(OH)D. Details of the laboratory methodology, quality control protocol can be found in the Laboratory Method manuals^54^.

### Covariates

We measured a number of covariates, including age, gender, race/ethnicity, marital status, education, household income, self-perceived health condition, smoking and drinking status, physical activity, sleep duration, body mass index (BMI), and time of blood draw. NHANES additionally measured vitamin D supplement. The phrasing of many questions in CLHLS and NHANES surveys were not identical, but were able to obtain measurements for each category. Missing values of covariates were reported separately (ranging from 0.1%-9% in both datasets).

In CLHLS, age was calculated as the difference between the interview dates and birth dates, verified through family members, genealogical records, ID cards, and household registration booklets. In NHANES, age was asked and recorded at the time of the screening. Individuals who were 80 years and over were topcoded as 80 years of age. In CLHLS, we coded ethnicity as Han Chinese and ethnic minorities, while in NHANES, race/ethnicity was categorized into Mexican American, other Hispanic, non-Hispanic White, non-Hispanic Black, non-Hispanic Asian, and other races. Years of schooling of CLHLS participants were divided into two groups: formal education (> = 1 year education), and no formal education. In NHANES, educational level was divided into five categories: less than 9^th^ grade, 9–11^th^ grade, high school graduate/GED or equivalent, some college or AA degree, college graduate or above. In CLHLS, annual household income of one year before the interview year was recorded, and categorized into tertiles. In NHANES, poverty income ratio (PIR) for the household, which is the ratio of total family income to the poverty threshold for the year of the interview, was used to represent income level, and was divided into low income (0–1.85), middle income (1.86–3.50), and high income (>3.51). In both CLHLS and NHANES, we defined marital status as married, separated, divorced, widowed, and never married, and self-perceived health condition as very good, good, fair, bad, and very bad.

In CLHLS and NHANES, smoking and drinking behavior were coded as “never”, “past” and “current” based on their answers in questionnaires. In CLHLS, we assessed the status of physical activity by the question of “whether exercise or not”. In NHANES, physical activity was defined as having vigorous or moderate work/recreational activities, or walking or using bicycle in a typical week. In both CLHLS and NHANES, we divided sleep duration into <6 hours, 6 to 9 hours, and >9 hours. BMI is the body weight divided by the square of the body height (unit: kg/m^2^). We used WHO standard of BMI in both CLHLS and NHANES, which defined a BMI of <18.5 kg/m^2^ as underweight, a BMI of > = 18.5 to <25 kg/m^2^ as normal weight, a BMI of > = 25 to <30 kg/m^2^ as overweight, and a BMI of > = 30 as obese. Serum 25(OH)D has seasonal variation. The time of blood draw was recorded in months (May to September) in CLHLS, and in summer (May to October) or winter (November to April) in NHANES. Participants who reported taking vitamin D supplements 30 days prior to the survey were classified as having vitamin D supplements in NHANES.

### Statistical analysis

We used SAS, version 9.4 (SAS Institute Inc., Cary, NC) for all analyses. We summarized participants’ demographic and lifestyle characteristics using descriptive statistics. We reported the mean and SE (standard error) for continuous variables, sample size and proportion for categorical variables. Vitamin D status was dichotomized into non-deficiency (> = 50 nmol/L) and deficiency (<50 nmol/L). We used linear regression and logistic regression to predict serum 25(OH)D concentration, adjusted for month/season of blood draw, age, gender, race/ethnicity, marital status, education, household income, self-perceived health condition, smoking and drinking status, physical activity, sleep duration, BMI, and vitamin D supplement. Weight was applied in the analysis to reflect the sampling design of the CLHLS and NHANES^10^. We calculated coefficients, Odds Ratios (ORs), and 95% Confidence Intervals (CIs) to estimate the magnitude of predictors on serum 25(OH)D level. Results were considered significant at p < 0.05.

### Ethical approval

NHANES was approved by the US Center for Disease Control and Prevention (CDC) National Center for Health Statistics Ethics Review Board, and CLHLS was approved by the Institutional Review Board (IRB) at Peking University and Duke University; participants in both studies gave informed consents. All methods were performed in accordance with the relevant guidelines and regulations.

## Data Availability

The datasets generated and/or analysed during the current study are available in the NHANES website. https://wwwn.cdc.gov/nchs/nhanes/continuousnhanes/default.aspx?BeginYear=2005.
